# Synthetic MRI for stroke: a qualitative and quantitative pilot study

**DOI:** 10.1038/s41598-022-15204-8

**Published:** 2022-07-07

**Authors:** Joachim André, Sami Barrit, Patrice Jissendi

**Affiliations:** 1 Department of Radiology, Hôpital Erasme, Université Libre de Bruxelles (ULB), Route de Lennik, 808, 1070 Anderlecht, Brussels, Belgium; 2 Department of Neurosurgery, Hôpital Erasme, Université Libre de Bruxelles (ULB), Brussels, Belgium; 3 Department of Radiology, CHU Saint-Pierre, Brussels, Belgium

**Keywords:** Neuroscience, Neurology

## Abstract

Synthetic MR provides qualitative and quantitative multi-parametric data about tissue properties in a single acquisition. Its use in stroke imaging is not yet established. We compared synthetic and conventional image quality and studied synthetic relaxometry of acute and chronic ischemic lesions to investigate its interest for stroke imaging. We prospectively acquired synthetic and conventional brain MR of 43 consecutive adult patients with suspected stroke. We studied a total of 136 lesions, of which 46 DWI-positive with restricted ADC (DWI + /rADC), 90 white matter T2/FLAIR hyperintensities (WMH) showing no diffusion restriction, and 430 normal brain regions (NBR). We assessed image quality for lesion definition according to a 3-level score by two readers of different experiences. We compared relaxometry of lesions and regions of interest. Synthetic images were superior to their paired conventional images for lesion definition except for sFLAIR (sT1 or sPSIR vs. cT1 and sT2 vs. cT2 for DWI + /rADC and WMH definition; *p* values < .001) with substantial to almost perfect inter-rater reliability (κ ranging from 0.711 to 0.932, *p* values < .001). We found significant differences in relaxometry between lesions and NBR and between acute and chronic lesions (T1, T2, and PD of DWI + /rADC or WMH vs. mirror NBR; *p* values < .001; T1 and PD of DWI + /rADC vs. WMH; *p* values of 0.034 and 0.008). Synthetic MR may contribute to stroke imaging by fast generating accessible weighted images for visual inspection derived from rapidly acquired relaxometry data. Moreover, this synthetic relaxometry could differentiate acute and chronic ischemic lesions.

## Introduction

Stroke care relies on timely and proper imaging. In the acute stage, it guides therapeutic interventions such as intravenous thrombolysis, endovascular treatments, and neurosurgery by early detection of acute ischemic stroke and intracranial hemorrhage (ICH) and characterization of infarct core penumbra and cerebral circulation. In the subacute or chronic stages, it supports the clinician’s diagnosis, management, and prognosis by describing lesion evolution and associated brain abnormalities. As MRI performance for these purposes is well substantiated, its scanning duration remains a drawback^1,2^. Along these lines, emerging techniques such as synthetic MR imaging (sMRI) are promising by their potential to provide fast single-acquisition of quantitative multi-parametric data about tissue properties using a multi-dynamic multi-echo sequence. This sequence repeats the same gradient reversal process used to create a single gradient echo to produce additional gradient echoes after a unique radiofrequency pulse^3,4^. Synthetic weighted images may be generated by virtually adjusting parameters such as echo, repetition, and inversion delay times, using mathematical inferences rather than being predetermined by multiple acquisitions of different sequences as in conventional MR imaging (cMRI). Different uses of sMRI in neuroimaging were reported for gliomas, brain metastasis, and multiple sclerosis^5–9^. To our knowledge, only two recent studies investigated sMRI application for stroke. Duchaussnoy et al. reported a specific interest in synthetic T2 mapping to gauge wake-up stroke onset time^10^. Li et al. provided a phantom validation study of the reliability of sMRI relaxometry measures for chronic ischemic stroke^11^. Hence, no study has specifically addressed overall sMRI clinical applicability for stroke. Therefore, we compared synthetic and conventional paired images and investigated synthetic relaxometry for acute and chronic ischemic lesions in controlled clinical conditions.

## Methods

### Study population

The institutional review board (*Comité d’éthique du CHU Saint-Pierre*) approved the study and waived the need for informed consent. This study was performed in accordance with relevant guidelines and regulations. We prospectively enrolled a total of 45 consecutive adult patients (aged 18 or more) admitted for sudden onset of neurological deficit or symptoms highly suggestive of stroke from January 2017 to March 2018. Our inclusion criteria were (i) the conclusion of the early management (i.e. at least 24 h after their admission) with (ii) no therapeutic interventions (i.e., intravenous fibrinolysis, endovascular treatment, or neurosurgery) and (iii) no acute ICH diagnosed. We performed a dedicated MRI session comprising conventional and synthetic acquisitions for this purpose. We observed four technical incidents: two patients presented major motion artifacts hindering conventional image interpretation, and two did not undergo the synthetic acquisition due to human operator error. A diagram illustrates the flow of participants through the study (Fig. S1) and the demographic data are given (Table S1) in Supplemental Material.

### MRI acquisition

The whole patient group was scanned on a 1.5 T MR imaging scanner (GE Healthcare, Chicago, IL) using a 24-channel head coil. Institutional cMRI stroke protocol includes 2D axial T1, FLAIR, T2*, DWI and apparent diffusion coefficient (ADC) mapping, coronal PROPELLER T2, and 3D TOF sequences. A 2D axial multi-dynamic multi-echo acquisition was then performed to provide the quantitative mapping of T1, T2, and PD using MAGnetic resonance image Compilation (MAGiC, GE Healthcare) post-processing software^5^. Synthetic images generated by MAGiC based on longitudinal R1 relaxation rate, transverse R2 relaxation rate, and PD maps have been adapted to maximize tissue contrast. The total protocol duration was 24 min (17 min 20 s for the conventional protocol and 6 min 40 s for the synthetic acquisition). The acquisition details are given (Table S2) in Supplemental Material.

### Ischemic lesions definitions

**Figure 1:**
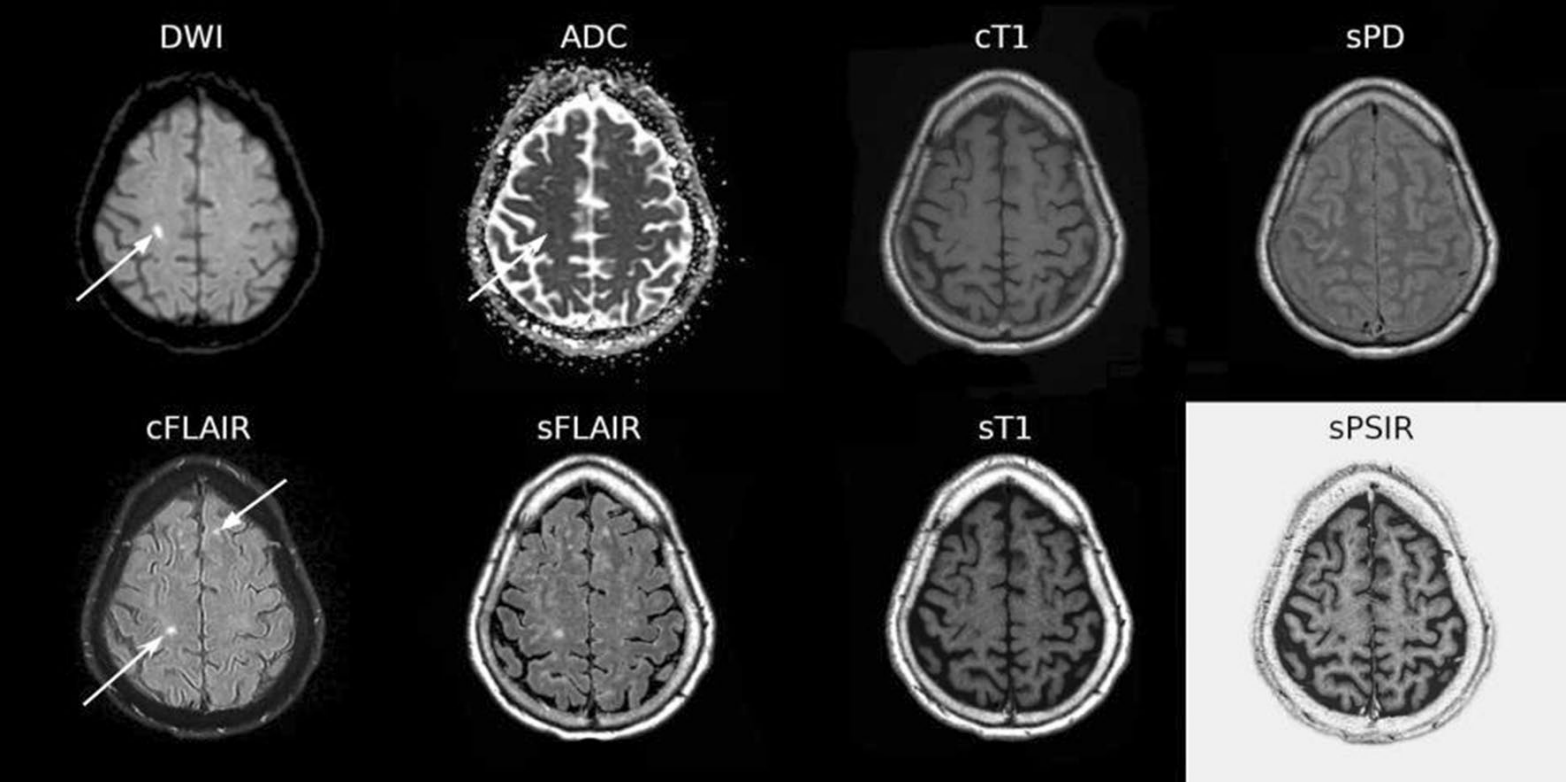
Axial conventional (DWI, ADC, cFLAIR, cT1) and synthetic (sFLAIR, sT1, sPD, sPSIR) weighted images depicting acute (DWI + /rADC, arrowhead) and chronic (WMH, arrow) ischemic lesions.

DWI-positive with restricted ADC (DWI + /rADC)—i.e., appearing bright on DWI and dark on ADC maps—or white matter T2/FLAIR hyperintensities not meeting the previous criteria (WMH)—i.e., appearing bright on T2/FLAIR images but not dark on ADC maps—were defined based on the cMRI images (Fig. 1) by consensus between an experienced neuroradiologist (PJ) and a neuroradiologist trainee (JA) and mapped in a common data collection sheet. For this purpose, DWI + /rADC and WMH were referred to as acute and chronic ischemic lesions respectively.

### Qualitative analysis

We performed the qualitative analysis for the first 25 consecutive eligible patients. The two reviewers assessed weighted image quality independently, randomly, and blinded to the clinical information and other reviewer assessment after a minimal 2-week memory-washout period of the consensus mentioned above on PACS consoles using the same settings for image display. We collected the qualitative assessment of lesions visibility and definition using a data collection sheet according to a 3-level score for each sequence of cMRI (cT1, cT2, cFLAIR) and sMRI (sT1, sPSIR, sT2, sFLAIR) as 1 (no lesion visible), 2 (lesion visible), 3 (well-defined lesion) for acute and chronic ischemic lesions. Finally, we compared the two individual data collection sheets of qualitative assessments with the common data collection of the consensus.

### Quantitative analysis

**Figure 2:**
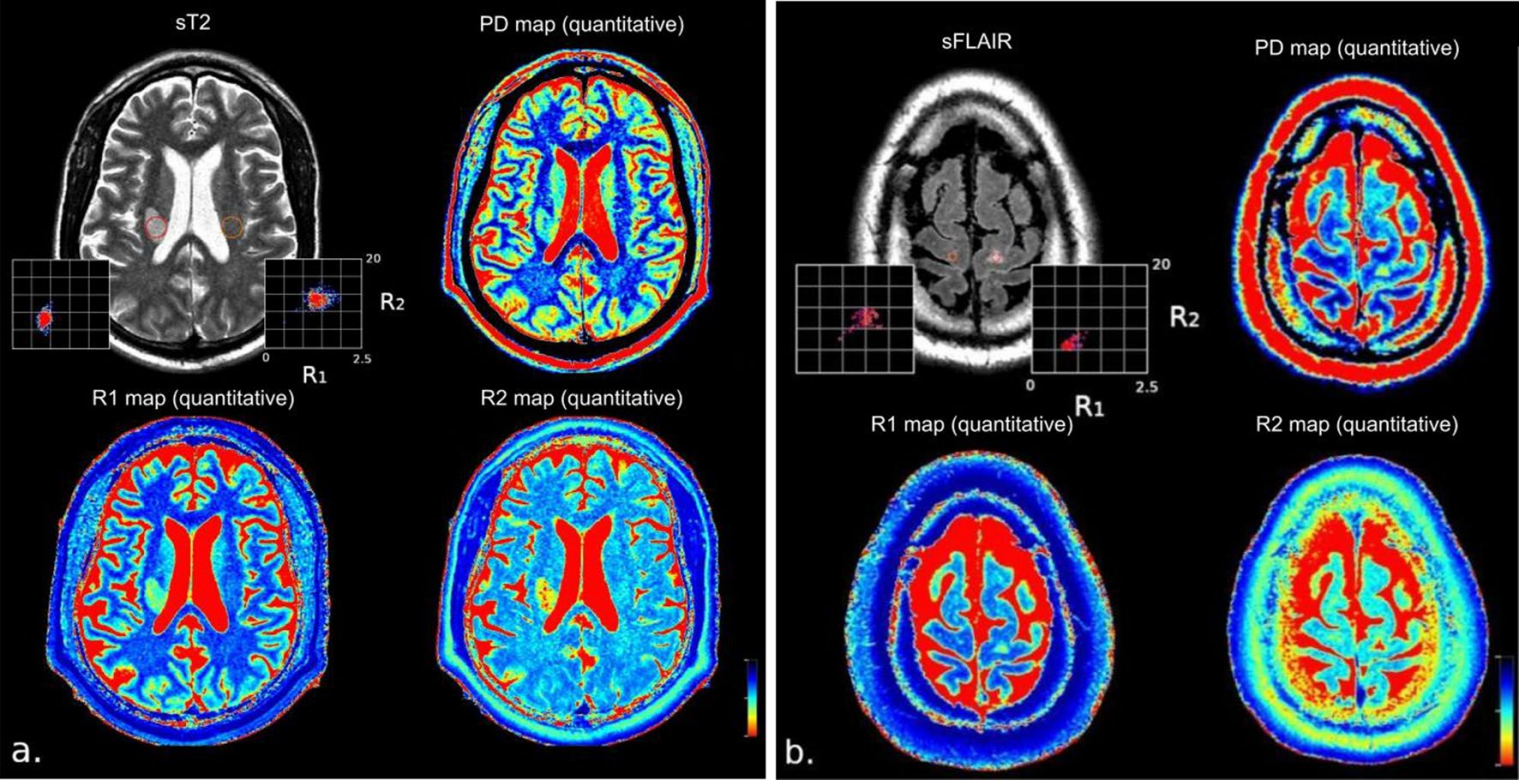
Representative examples of ROI in lesions (**a** acute ischemic lesion DWI + /rADC, **b** chronic ischemic lesion WMH) and contralateral mirror NBR in a sT2 weighted image (upper left quadrant) with R_1_R_2_ Plot (scales: 0–2.5 [1/s]; 0–20 [1/s]) and R1 (lower left quadrant, red-to-blue colorbar scale: 0–2000 [ms]), PD (upper right quadrant, red-to-blue colorbar scale: 0–100 [pu]) and R2 (lower right quadrant, red-to-blue colorbar scale: 0–200 [ms]) quantitative maps.

A single investigator (JA) performed the quantitative data collection on MAGiC on the 43 eligible patients. We delineated a circular ROI of DWI + /rADC and WMH lesions aiming to maximize the ROI-lesion overlap while not including perilesional tissue on the synthetic images based on a visual matching with the cDWI and cFLAIR images. Then, we placed a circular ROI of the same size in the contralateral apparent normal brain region (NBR) for each lesion in the same section and location, so-called the mirror ROI (Fig. 2). For WMH, we considered only the most representative lesion—i.e., the most extensive lesion presenting a homogeneous hypersignal in cFLAIR with an adequate mirror ROI per patient. The white matter areas showing an overlap between acute and chronic lesions were not considered, as well as lacunar infarcts, because of their heterogeneity or a small size (millimetric), jeopardizing consistent relaxometry measurements. We manually placed ten ROIs in different NBR structures: superficial and deep white matter (WM), grey matter (GM), and cerebrospinal fluid (CSF). The superficial and deep WM structures were corresponding to the left and right centrum semiovale, and, genu and splenium of the corpus callosum; the superficial and deep GM structures corresponding to the left and right superior frontal cortex and thalami; the CSF structures corresponding to the left and right anterior horns of the lateral ventricles. We collected R1, R2, and PD values. The works of Blystad et al. inspired this approach^3^.

### Statistical analysis

We proceeded to an exploratory data analysis approach and no correction for the multiple comparisons problem was performed. We compared paired or independent ordinal variables or dichotomous dependent variables by nonparametric tests of Wilcoxon signed-rank, Mann–Whitney–Wilcoxon, or McNemar's test, respectively. The inter-rater reliability was measured by quadratic Cohen's weighted kappa coefficient (κ). We calculated the Kendall rank correlation coefficient (τ) for the ordinal association. Statistical significance was predetermined as a *p* value < 0.05 with preferred two-tailed tests. We computed statistics and data visualization on the open-source R, Version 4.0.3 (R statistical and computing software; http://www.r-project.org/)—all our scripts are available on a dedicated github repository and Supplemental Material.

### Ethical approval

The data and code are fully available upon request for reviewers to test reproducibility. About ethical considerations, the institutional review board approved this study while waiving consent to participate/for publication. We certify that the submission is original work and is not under review at any other publication.

## Results

### Study population

The 43 patients included were 16 females (age range 25–84 years, mean age 54 years) and 27 males (age range 22–89 years, mean age 63 years), with a combined total of 136 lesions of which 46 DWI + /rADC and 90 WMH—4 patients being free of diagnosed lesions, and, 430 NBR. We did not find any DWI/FLAIR mismatched lesion of potentially undiagnosed silent hyperacute stroke or T2* hypointensity of an acute ICH.

### Qualitative analysis

**Figure 3:**
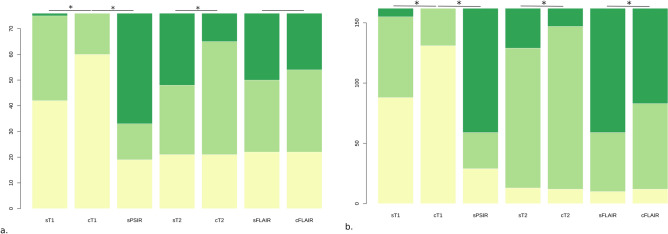
Stacked bar plot of overall image quality for conventional and synthetic MR imaging for (**a**) 46 acute (DWI + /rADC) and (**b**) 90 chronic (WMH) ischemic lesions in the first 25 consecutive patients. Each contrast-weighted image quality for lesion definition and visibility was rated according to a 3-level score by two readers as: 1—no lesion visible (yellow), 2—lesion visible (light green), 3—well-defined lesion (green). Significant differences (*p* < 0.05) between weighted images are indicated by an asterisk.

The assessed quality of synthetic images was significantly superior to their paired conventional images for acute and chronic ischemic lesions except for sFLAIR (sT1 or sPSIR vs. cT1 and sT2 vs. cT2 for DWI + /rADC and WMH definition; *p* values < 0.001). Indeed, sFLAIR qualitative comparison was only significantly superior for chronic ischemic lesions (sFLAIR vs. cFLAIR for DWI + /rADC and WMH definition, *p* values of 0.49 and < 0.001). The readers reported a higher noise level and a greater number of artifacts consisting of thin and scattered hyperintensities predominantly at the parenchymal interface giving an overall granulated aspect of sFLAIR and resulting in lower overall image quality than cFLAIR (Fig. S2). The proportion of visible lesions on sT1 and sPSIR was significantly superior for acute and chronic ischemic lesions (sT1 or sPSIR vs. cT1 for DWI + /rADC and WMH visibility, *p* values < 0.001). A grouped and stacked barplot displays these results (Fig. 3). All inter-rater reliability measures were significant with the lowest observed for cT2 and the highest for cFLAIR (κ = 0.47 [0.29–0.66] and 0.93 [0.89–0.98], *p* values < 0.001, Table 1). Tables display these results (Tables S3 and S4) in Supplemental Material.

**Table 1:** Inter-rater reliability (PJ vs JA).

	Flair		T1		T2		PD		PSIR		Diffusion	
	k	*p*	k	*P*	k	*P*	k	*P*	k	*P*	k	*p*
Synthetic	0.896 [0.835; 0.956]	< 0.001^†^	0.767 [0.672; 0.862]	< 0.001^†^	0.711 [0.572; 0.851]	< 0.001^†^	0.905 [0.839; 0.971]	< 0.001^†^	0.932 [0.89; 0.974]	< 0.001^†^		
Conventional	0.934 [0.888; 0.981]	< 0.001^†^	0.84 [0.744; 0.936]	< 0.001^†^	0.475 [0.293; 0.657]	< 0.001^†^					0.836 [0.609; 1]	< 0.001^†^
Overall	0.915 [0.878; 0.953]	< 0.001^†^	0.808 [0.707; 0.909]	< 0.001^†^	0.611 [0.501; 0.72]	< 0.001^†^						

PJ, experienced neuroradiologist; JA, neuroradiologist trainee; Cohen's κ coefficient—[95% CI].

^†^*p* < 0.001.

### Quantitative analysis

**Figure 4:**
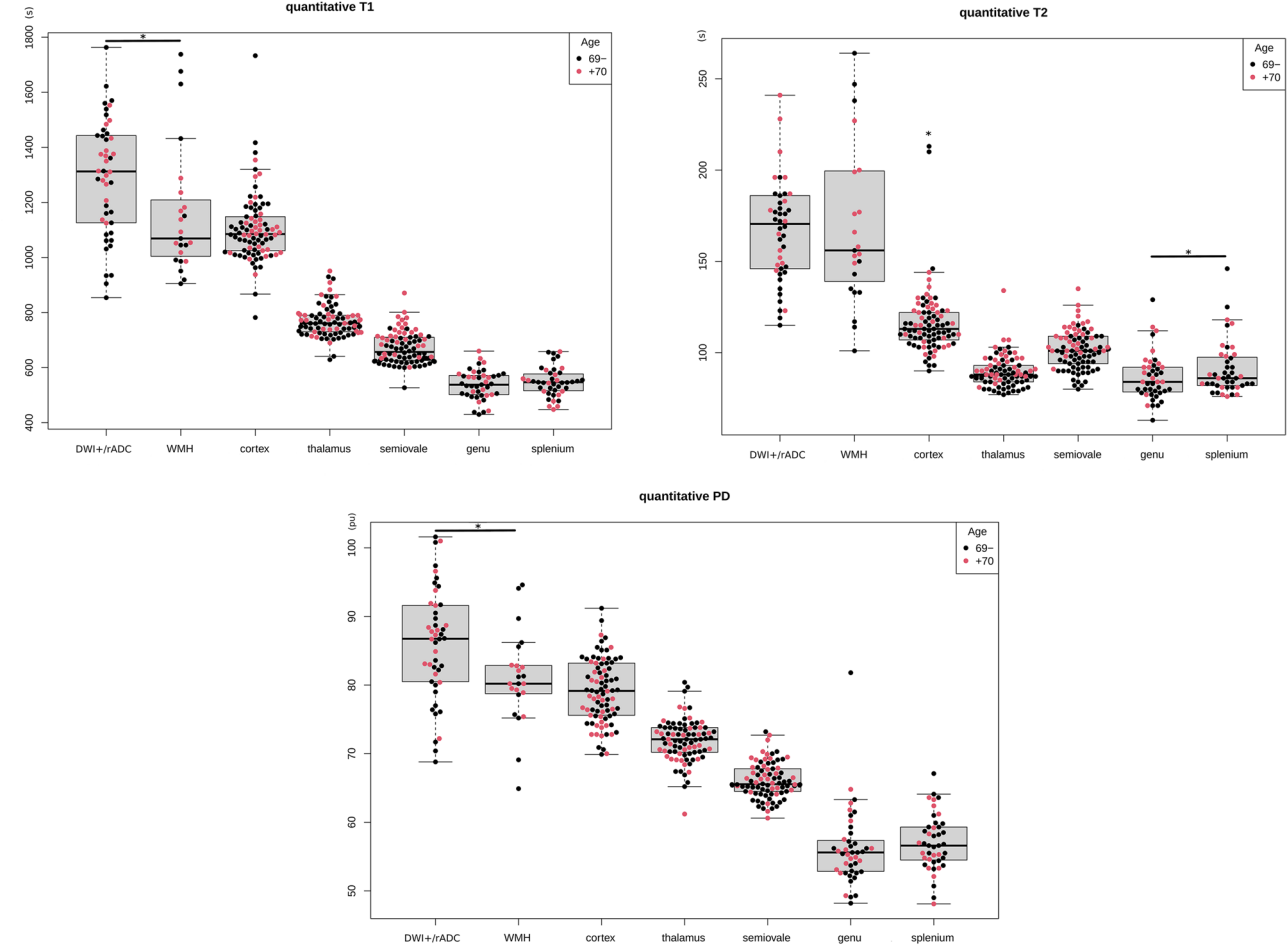
Beeswarm-box plots depicting quantitative synthetic findings of 46 acute (DWI + /rADC) and 23 chronic (WMH) ischemic lesions, and, 430 NBR. Significant differences (*p* < 0.05) between ROI are indicated by an asterisk.

All relaxometry measures of acute and chronic ischemic lesions compared to their mirror NBR were significantly different (T1, T2, and PD of DWI + /rADC or WMH vs. mirror NBR; *p* values < 0.001). Relaxometry measures of acute compared to chronic ischemic lesions were significantly different except for T2 (T1, T2, and PD of DWI + /rADC vs WMH; *p* values of 0.034, 0.697, and 0.008). Concerning NBR paired relaxometry measures, only the T2 of the right and left frontal cortex, and the genu and splenium of corpus callosum ROIs were significantly different (*p* values of 0.042 and 0.035). Age was rank-correlated with statistical significance for all relaxometry measures of CSF and centrum semiovale, T2 and PD of the thalamus, and T2 of cortex and genu of corpus callosum (absolute value of τ ranging from 0.16 to 0.42, *p* values from 0.036 to < 0.001). A beeswarm-box plot (Fig. 4) and tables display these results (Tables S5 and S6) in Supplemental Material.

## Discussion

Due to scarce evidence available, a full-scale study implementing sMRI in the management of stroke patients from the outset was considered premature. Therefore, this pilot study aimed to investigate the interest of sMRI for ischemic stroke in controlled clinical conditions. Of the 45 consecutive patients enrolled, four were concerned by technical incidents. None were due to the synthetic acquisition but two related to it as human operator errors. Specific training and experience combined with dedicated MRI protocols for clinical application should prevent these. The remaining two technical incidents were major motion artifacts only compromising conventional images.

### Qualitative analysis

The quality of synthetic images was assessed superior to conventional weighted images for DWI + /rADC and WMH definition—sFLAIR not reaching significance, particularly sT1 and sPSIR compared to cT1 for acute and chronic ischemic lesions. The interest of sPSIR has been previously reported for multiple sclerosis lesions detection and intracranial vessel characterization. To our knowledge, no previous study has evaluated its interest in stroke. Substantial to almost perfect inter-rater reliability was obtained for synthetic imaging between the two observers but lower than conventional imaging, except for the cT2. Both readers were naive to sMRI, which may contribute, combined with a significant difference in experience, to the overall lower inter-individual agreement observed for synthetic images. However, the lowest inter-rater reliability concerned a conventional weighted-image (cT2). This notable exception may be due to a coronal acquisition thought to be more difficult to interpret for inexperienced readers. Interestingly, this suggests that the specification of the cMRI acquisition protocol may have more effect on inter-reader agreement than the synthetic generation of weighted images. According to the readers’ feedback, an sFLAIR had lower overall image quality than cFLAIR due to noise and artifacts. However, this subjective assessment was not associated with a significant difference in image quality ratings for lesions definition and visibility on FLAIR images for inter-method (synthetic versus conventional) and inter-readers qualitative assessments (experienced neuroradiologist versus trainee) comparisons. This supports easily detectable artifacts not hindering lesion characterization even for inexperienced readers in line with previous findings of Ryu et al.^12^ Moreover, deep learning has been recently used to improve the quality of synthetic FLAIR images with promising results^13^.

### Quantitative analysis

Acute and chronic ischemic lesions relaxometry profiles compared to their mirror NBR showed higher lesional T1, T2, and PD. Interestingly, a comparison between acute versus chronic ischemic lesions profiles retrieved significant differences for T1 and PD but not T2. Experimental studies on rodent models undergoing middle cerebral artery occlusions have previously outlined for subsequent ischemic lesions a maximization of T1 and T2 relaxation times and PD at 24–48 h to decline between 4 and 7 days—presumed to reflect the development of vasogenic edema and increasing tissue water content^14–16^. Accordingly, the synthetic quantitative profiles retrieved in our study seem consistent—with the exception of paradoxical but not significantly lower T2 of acute lesions. However, the acute and chronic stroke groups defined in this study presented heterogeneous relaxometry values (as depicted in Fig. 4) that may be due to high variability in lesion age and topography. Therefore, no definitive conclusion can be drawn in absence of precise onset-time and histopathological correlations with limited sample size. Along these lines, Duchaussoy et al. reported a correlation between synthetic T2 mapping and stroke onset-time, supporting it as a potential biomarker of interest for the management of patients with stroke of unknown onset^10^. Overall quantitative findings of NBR were in line with the literature, however variations in populations studied and methods of segmentation of ROIs (automated, semi-automated, or manually drawn) and technology (e.g., quantitative cMRI, sMRI, or MRI fingerprinting) to acquire the relaxometry may contribute to differences observed^17–21^. Interestingly, we found a significant difference in T2 relaxometry between left and right frontal cortex ROIs. We postulate a left–right asymmetric bias due to the manual segmentation method used^22^. This asymmetric bias may have only reached significance for T2 of cortex ROIs due to cortico-subcortical atrophy (associated with the partial volume effect of CSF)^23^ of the overrepresented elderly patients in the study population. In this study, T1, T2, and PD of WM values increased with age which is consistent with the recent findings of Hagiwara et al^24^. Our nonsignificant finding for the grey matter's PD decreasing with age may corroborate the previous hypothesis postulated about the potential left–right asymmetric segmentation bias due to the manual segmentation method used, partial volume effect, and population studied.

### Limits

As a pilot study, only selected patients—i.e., after the conclusion of their early management and clinical stability ensured—were recruited in a dedicated MRI session. Our study was not designed to evaluate sMRI diagnosis performance nor its clinical significance—i.e. no clinical correlation was performed. Indeed, we assessed lesions definition and visibility based on a consensus. Moreover, we used DWI + /rADC and WMH as surrogate markers of acute and chronic ischemic lesions. However, this approach is limited by this simplistic categorization of two heterogeneous groups including different etiopathogenic types of ischemic lesions. Also, as ischemia is a widely accepted cause of white matter changes, clearly not all such lesions are due to it. Furthermore, its small sample size prevented complementary analyses, such as image quality depending on lesion topography. Finally, we can not exclude confirmation bias from the potential inclination towards a positive assessment of sMRI.

### Perspectives

A full-scale study of sMRI for stroke would be the next step to confirm its clinical applicability and significance. The sMRI may fastly provide surrogate cMRI images and unique information for stroke care. Indeed, sMRI can offer versatile relaxometry weighting parameters dynamically adjustable for contrast and color-coded visualization. Moreover, the synthetic quantitative data may be particularly suitable for computational analysis to develop lesion automated detection algorithms (e.g., of hyperacute ischemic lesion or ICH)^25^, define biomarkers (e.g., of stroke salvageability, onset time, or topography)^10^, and, constitute voxel-wise stroke-specific atlases^26^. However, sMRI diagnostic accuracy and time efficiency for specific stroke imaging endpoints will have to be determined in clinical settings comprising the critical time window of acute stroke and compared to current criterion standards (e.g. multimodal MRI) and other MR rapid acquisition techniques.

## Conclusion

An MRI protocol combining conventional and synthetic acquisitions was performed on 43 consecutive stroke patients in controlled clinical conditions. The qualitative analysis of synthetic MRI found superior overall performance for acute and chronic ischemic lesions definition compared to conventional MRI. Moreover, the inter-rater reliability between two readers with different experiences supports the accessibility of this technique. The quantitative analysis of synthetic relaxometry found significant differences in profiles of acute and chronic ischemic lesions and consistent findings of distinct normal brain regions with the literature suggesting promising interest in this approach for stroke. Therefore, a full-scale study of synthetic MRI diagnosis performance in clinical settings comprising the critical time window of acute stroke early management would be the next step to investigating its clinical significance. Moreover, the synthetic quantitative data may define useful stroke biomarkers for future radiomics applications.

## Supplementary Information

Supplementary Figures.

Supplementary Tables.

Supplementary Information 3.

## Abbreviations

sMRI: Synthetic MR imaging
cMRI: Conventional MR imaging
ICH: Intracranial hemorrhage
PD: Proton density
MAGiC: MAGnetic resonance image Compilation
DIR: Double inversion recovery
PSIR: Phase-sensitive inversion recovery
NBR: Normal brain region
κ: Cohen's kappa coefficient
τ: Kendall’s tau rank correlation coefficient
pu: Percentage unit
