# Supplementary material for: Synthetic MRI for stroke: a qualitative and quantitative pilot study
Source: Sci Rep. 2022 Jul 7;12:11552. doi: 10.1038/s41598-022-15204-8 (PMC9262877; doi:10.1038/s41598-022-15204-8)
Supplement: Supplementary file 1 — Supplementary Figures. [file 41598_2022_15204_MOESM1_ESM.pdf]

Supplemental figures - miniatures and legends

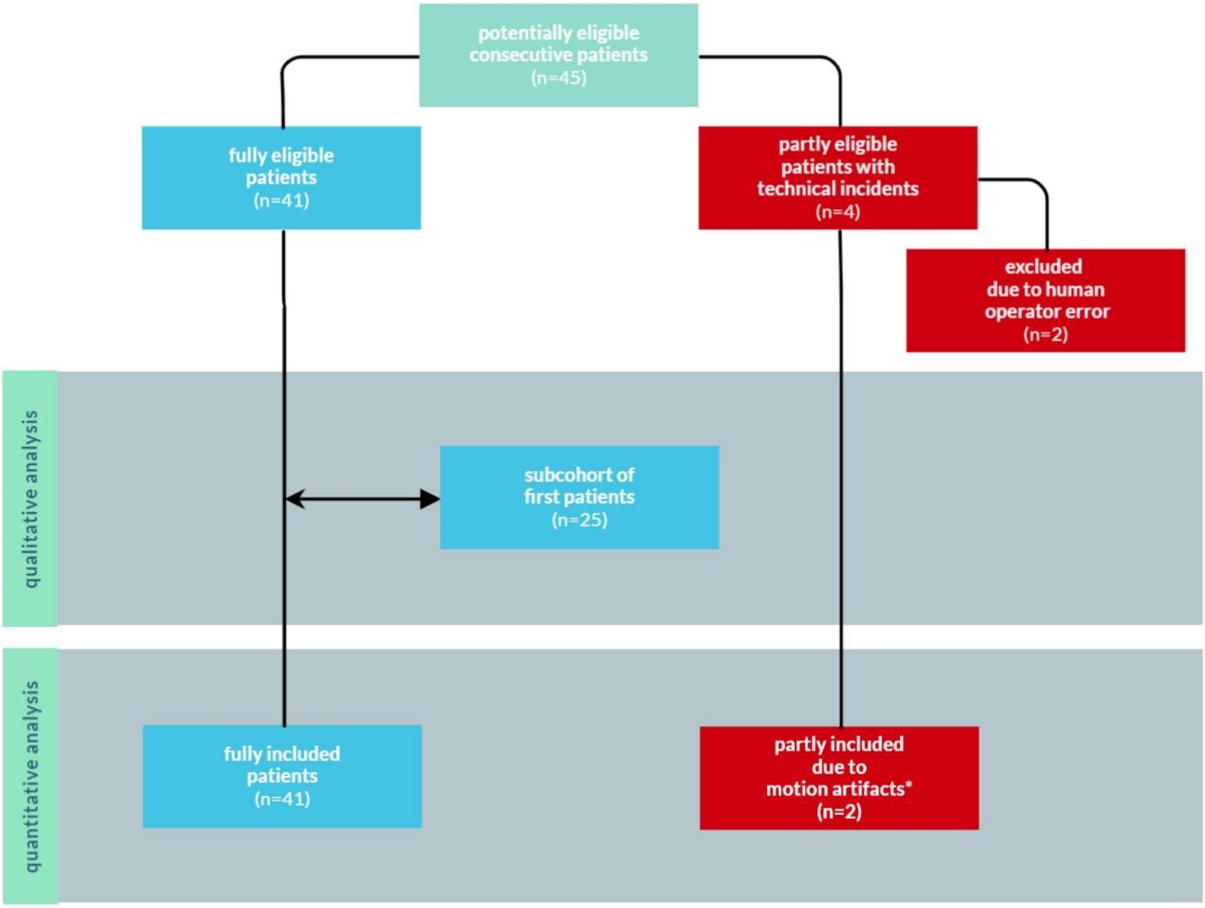

Fig S1. Flow chart diagram of participants through the study: only the first fully eligible 25 consecutive patients were included in the qualitative analysis; as human operator error consisted of absence of synthetic acquisition, the two patients concerned were excluded from any analysis; as motion artifacts only compromised conventional acquisitions, the two patients concerned were included in the quantitative analysis.

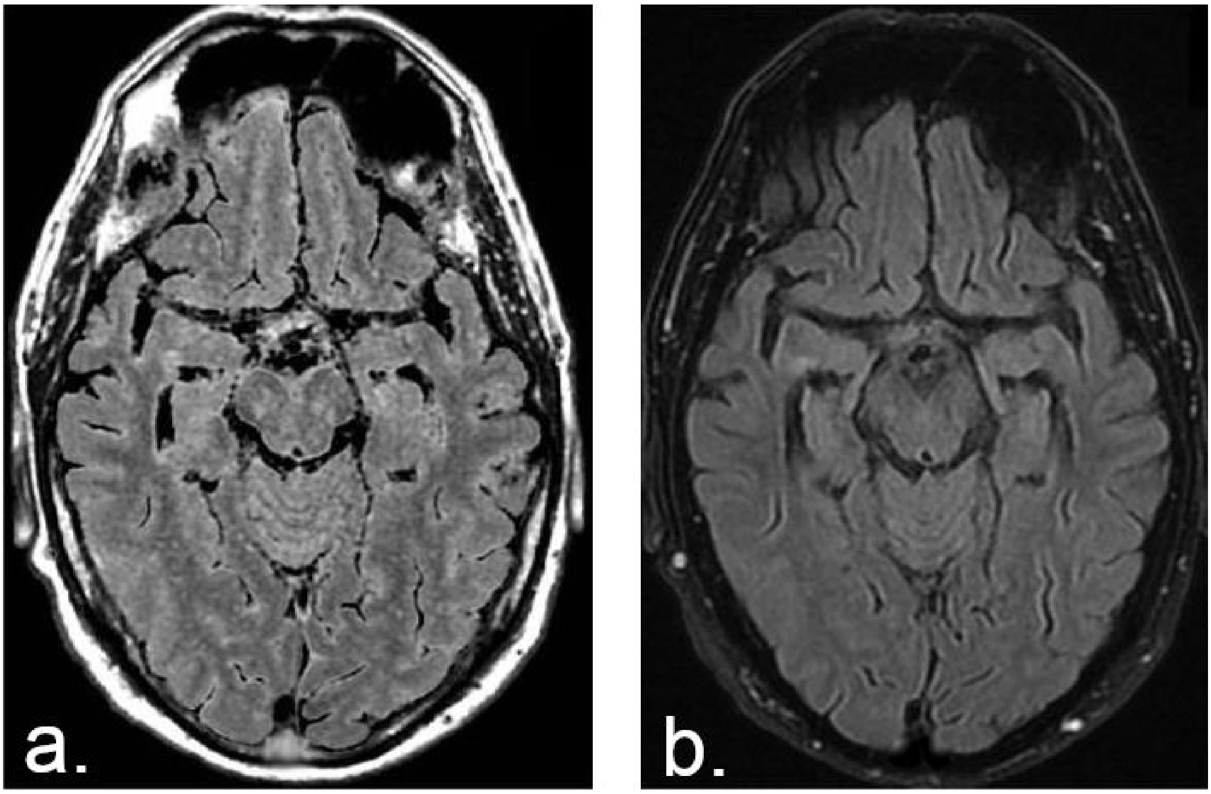

Fig S2. Axial synthetic (a) and conventional (b) FLAIR weighted-images; note the thin and scattered artifact hyperintensities predominant at parenchymal interface giving an overall granulated aspect and lower image quality of FLAIR synthetic images.
